# Implementing resources to support the diagnosis and management of Chronic Fatigue Syndrome/Myalgic Encephalomyelitis (CFS/ME) in primary care: A qualitative study

**DOI:** 10.1186/s12875-016-0453-8

**Published:** 2016-06-04

**Authors:** Kerin Bayliss, Lisa Riste, Rebecca Band, Sarah Peters, Alison Wearden, Karina Lovell, Louise Fisher, Carolyn A Chew-Graham

**Affiliations:** Public Programmes, Central Manchester University Hospitals NHS Foundation Trust, University of Manchester, Manchester Academic Health Science Centre, Manchester, UK; Centre for Primary Care, Institute of Population Health, University of Manchester, Manchester, UK; School of Psychology, University of Southampton, Southampton, UK; School of Psychological Sciences, University of Manchester, Manchester, UK; School of Nursing, Midwifery and Social Work, University of Manchester, Manchester, UK; National School for Primary Care Research, University of Manchester, Manchester, UK; Primary Care and Health Sciences and National School for Primary Care Research, Keele University, Keele, Staffordshire UK

**Keywords:** Chronic fatigue syndrome (CFS), Myalgic Encephalomyelitis (ME), Resources, Training, Implementation, Qualitative research, Primary health care

## Abstract

**Background:**

Previous research has highlighted that many GPs lack the confidence and knowledge to diagnose and manage people with CFS/ME. Following the development of an online training module for GPs, and an information pack and DVD for patients, this study explored the extent to which these resources can be implemented in routine primary care.

**Methods:**

Semi structured qualitative interviews were completed with patients and GPs across North West England. All interviews were transcribed and analysed using open exploratory thematic coding. Following this thematic analysis, the authors conducted a further theory-driven analysis of the data guided by Normalisation Process Theory.

**Results:**

When used in line with advice from the research team, the information resource and training were perceived as beneficial to both patients and GPs in the diagnosis and management of CFS/ME. However, 47 % of patients in this study did not receive the information pack from their GP. When the information pack was used, it was often incomplete, sent in the post, and GPs did not work with patients to discuss the materials. Only13 out of 21 practices completed the training module due to time pressures and the low priority placed on low prevalence, contentious, hard to manage conditions. When the module was completed, many GPs stated that it was not feasible to retain the key messages as they saw so few patients with the condition. Due to the complexity of the condition, GPs also believed that the diagnosis and management of CFS/ME should take place in a specialist care setting.

**Conclusion:**

While barriers to the implementation of training and resources for CFS/ME remain, there is a need to support CFS/ME patients to access reliable, evidence based information outside primary care. Our findings suggest that future research should develop an online resource for patients to support self-management.

**Electronic supplementary material:**

The online version of this article (doi:10.1186/s12875-016-0453-8) contains supplementary material, which is available to authorized users.

## Background

Chronic Fatigue Syndrome (CFS) or Myalgic Encephalomyelitis (ME) is characterized by disabling, unexplained fatigue that is not alleviated by rest and lasts at least four months. Symptoms can include post exertional malaise, unrefreshing sleep, weakness, pain, sore throat, headaches and concentration or memory problems. The NICE guidelines for CFS/ME emphasise the need for an early diagnosis in primary care with management tailored to patient needs [1]. However, some GPs see CFS/ME as a contentious illness while others report a lack of confidence and knowledge in how to diagnose and manage the condition, and how to refer patients to specialist services [2–5]. Furthermore, the condition is of low prevalence and low priority as it is not within the Quality and Outcome Framework (QOF) [6]. This has led to dissatisfaction with current practice among patients who tend to disengage from primary care [7, 8].

As part of the METRIC (ME Education, Training and Resources In Primary Care) study [3] we developed resources for practitioners and patients to support the diagnosis and management of CFS/ME in primary care. This included an online training module for GPs (available on the Royal College for General Practice website) and a resource pack for patients. The resource pack included information sheets on how to manage the main symptoms of CFS/ME. These were designed to be discussed within a consultation, enabling the patient to work with their GP to prioritise and manage their symptoms. A DVD giving advice from CFS/ME specialists and case studies of patient and carer experiences was also provided for the patient and their families to watch at home. The resources were based on patient and practitioner need and were informed by 44 qualitative interviews with GPs, patients, carers and CFS/ME specialists [3]. Patient and Public Involvement (PPI) was also central to the development of these resources and the research team included a patient co-applicant and a carer representative. Two patient involvement groups (comprising six members at sites in Bolton and Preston) were also consulted quarterly to inform the design and content of the resource [9].

The study presented a unique opportunity to explore the extent to which CFS/ME training and resources can be implemented in routine primary care, leading to a better understanding of the barriers and facilitators to the adoption and integration of new practices associated with medically unexplained conditions. The use of theoretical models of implementation is recommended to enable researchers to identify the conditions necessary for interventions to be successfully adopted in routine primary care [10]. The Normalization Process Theory (NPT), which uses four constructs to explore the dynamics of implementing, embedding, and integrating a new technology or complex intervention (Box 1) informed the analysis and helped us to understand the processes involved and work required for the implementation and sustainability of CFS/ME training and resources in primary care.

## Methods

### Ethics

Ethical approval was obtained from the Greater Manchester East Ethics Committee (10/H1001/5). R&D approval was granted by NHS Manchester, Central Lancashire, Stockport, Salford, Trafford, Bolton and Bury.

### Design

This qualitative study used individual face-to-face semi-structured interviews to collect data about engagement in the study and implementation of the resources.

### Sampling

#### Practitioners

One hundred and nine practices were approached to provide a purposive sample of 21 GP practices from seven PCTs in North West England. Practices were recruited using a variety of methods, including personal invitation (four PCTs), identification of practices with 10 or more patients with CFS/ME (one PCT), an introduction at two GP education meetings in the North West of England, and support from the North West Primary Care Research Network (one PCT). Five practices subsequently withdrew from the study (Table 1). A flyer outlined the aims of the study and gave the research team’s contact details for those who wanted further information. We attempted to sample on size of practice, demographics (inner city/urban/sub-urban; ethnic mix) and a mix of training/non-training practices. Each practice received £200 for participation.

**Table 1:** Number of GPs trained and patients recruited from each practice

Practice	Status	GP training (face to face)	GPs training (e-learning)	CFS/ME patients identified	n patients recruited to the study
1		0	1	7	3
2		0	1	36	5
3		0	0	4	2
4		0	1	7	0
5		0	1	4	0
6		0	0	3	0
7		6	0	58	9
8	withdrew	0	0	0	0
9		0	1	4	2
10		0	0	44	1
11		6	0	27	4
12		0	2	17	4
13		0	4	44	3
14	withdrew	0	0	0	0
15		0	0	28	6
16		0	1	28	8
17	withdrew	0	0	27	0
18		0	1	13	4
19	withdrew	1	0	0	0
20		0	2	6	6
21	withdrew	0	0	0	0
		13	15	357	57

All GPs in participating practices were given access to an online CFS/ME training module (hosted by the Royal College of General Practitioners (RCGP) website). Face-to-face training was offered where practices preferred delivery via another route. Each GP who completed the training received £50 (to a maximum of £300 per practice). Patient resource packs were delivered to all practices for use in consultation with new and existing CFS/ME patients [3]. The pack was designed for the patient to prioritise their symptoms and thereby create their own ‘personalised resource pack’ in conjunction with the GP.

#### Patients

Patients were recruited from participating GP practices. Searches of the practice databases were conducted by the practice manager to identify individuals with an existing diagnosis of CFS/ME. READ codes were supplied by the research team to assist with this process. GPs were asked to review these lists and to exclude patients with other conditions, or other factors that may account for their fatigue (e.g., cancer, recent bereavement). Patients were invited by a letter from the practice manager to take part in the study. This letter stated that the aim of the study was to improve the care of people with CFS/ME. An information sheet (available from the authors), reply slip and prepaid envelope were also enclosed. Patients sent this reply slip directly to the research team if they were interested in finding out more information about the study.

### Data collection

Six months following consent to take part, patients and GPs were invited by the researcher to take part in a face to face semi structured interview. Patients were interviewed in their own home, while GPs were interviewed at their practice. Not all interviewed GP had fully engaged in the training or research. All interviews were completed by KB.

Topic guides (Additional files 1 and 2) were developed from a review of the literature and research team (including patient and carer research partners) discussions. Patient interviews focused on their views on the CFS/ME patient resource and their experience with their GP before and after the practice had access to the online training. GP interviews explored their experience of managing people with CFS/ME before the participating in the study and their opinions on the training (face-to-face or online module) and the patient resource pack. All GPs were reimbursed for their time according to standard DH guidelines.

### Analysis

Interviews were digitally recorded and transcribed verbatim. Analysis was conducted in parallel with the interviews and was inductive, using components of thematic analysis [11]. Thematic categories were identified in initial interviews and then explored in subsequent interviews. Disconfirming evidence was actively sought and was used to modify emerging themes. Main categories were then compared across interviews and reintegrated into common themes [11]. Interview transcripts were read, annotated, and categorised independently by researchers of different professional backgrounds (Psychology, Primary Care, Nursing) and patient and carer research partners to increase reliability of the analysis [12]. Open coding was used rather than axial or selective coding initially [13]. It was agreed that theoretical saturation across the data sets was achieved when no new themes emerged during the final interviews.

Following familiarisation with the data through the initial thematic analysis, we conducted a further theory-driven analysis of the data guided by the four main constructs of NPT (Table 2) [14]. All researchers conducted this analysis separately, and the final analysis was agreed through discussion.

**Table 2:** NPT constructs (May and Finch, 2009)

NPT constructs	Description
1. Coherence	The meaning of the practice to actors – is there agreement on what the work is? e.g., Is there an understanding of the place of managing CFS/ME in primary care?
2. Cognitive participation	Engagement, individually and collectively, with the practice – is there agreement about who does the work? e.g., What kind of norms exists around who should manage CFS/ME? Is there a commitment to the management of CFS/ME in primary care?
3. Collective action	Interaction with pre-existing or established processes - is there agreement about how the work gets done? e.g., Are GPs using the resources and working together with patients to manage CFS/ME?, Is there formal or informal agreement about what works need to be done to achieve collaboration and what activities need to be performed to do it?
4. Reflexive monitoring	How the practice is assessed and understood by the actors – is there agreement on how to appraise the work? e.g., How is the impact of the training and resources evaluated?

## Results

Fifty-seven patients (21 % male and 79 % female, aged between 27 and 71 with a mean age of 46 years) were recruited from 13 practices (out of the 21 practices who had agreed to participate and sent letters out to patients: Table 1). Eleven of these patients (Table 3) and eight GPs participated in qualitative interviews.

**Table 3:** Patients interviewed at six-month follow-up

Patient ID	Sex	Age	Resources received	GP Appointment during follow-up period
7	Male	56	No	No
8	Female	43	No	Yes
11	Male	40	No	Yes^b^
18	Female	36	No	Yes^b^
21	Female	32	No	Yes^b^
24	Female	41	GP surgery^a^	Yes
25	Female	74	No	Yes^b^
35	Female	40	No	No
39	Female	65	GP	Yes^b^
42	Female	53	No	Yes
66	Female	27	GP surgery^a^	No

^a^Resource pack was received from the GP surgery in the post, or from a receptionist or nurse (not directly from the GP during a consultation)

^b^Patient had not discussed CFS/ME or the METRIC resources during consultations with the GP

GPs from ten practices completed the online training. A further three practices chose to receive face-to-face training delivered by the research team, representing 13/21 (62 %) participating practices (Table 1). Six out of the eight GPs interviewed had participated in the training, although not all had completed the online test and downloaded their completion certificate.

### Coherence: Understanding the place of primary care in the management of patients with CFS/ME

The research team experienced difficulty recruiting GP practices for training. Reasons given for a lack of engagement included a level of scepticism about CFS/ME, and the complexity of managing the condition and working with patients and their families:

*“not all doctors believe that there is Chronic Fatigue Syndrome and they just think it’s in the patient’s head.” (GPB01)*

*“[CFS/ME] is difficult, there are no easy answers. You know, why would you be interested in that? It doesn’t feel like there’s a big win for the doctor, I think.” (GPA05)*

*“It’s not attractive, I got forced in a way, going back right to the very beginning, by a belligerent husband, I had to do something, it didn’t matter what, do something… But, I do think the topic is off putting… it’s not easy going.” (GPA03)*

One GP highlighted a divide within their profession, with those who will manage patients with complex conditions such as CFS/ME, and those who prefer to refer on. There was an implication that, for some, the level of commitment required to manage patients over the longer term is too much for a primary care professional, and that CFS/ME should be managed in secondary care by specialists:

*“I think early on, doctors divide into those who feel that they can manage difficult things and those who feel that actually they are primary care physicians, so they should be doing primary work, and that things that are more difficult need somebody else to do it. And I remember a GP saying to me once, what’s the point of learning all these skills for picking up cues and eliciting people’s psychological problems, if you haven’t got a counsellor to refer them to? In other words, they didn’t see its management as their job… it is the test of stamina for the doctor, undoubtedly” (GPA05)*

Where GP practices were recruited to the study, there were barriers to subsequent patient recruitment which centred around the labelling of CFS/ME. GPs suggested that patients who had CFS/ME were not always coded on the practice computer system. This was due to a lack of clarity around the diagnosis and the fact that some GPs believed that the label of CFS/ME is stigmatizing:

*“I used to be very reluctant to make the diagnosis, because I thought it was quite stigmatising, and because I thought people did, kind of, act to the label; and I still think that does go on, actually (GPA05)*

*“it’s the confidence in being able to make a diagnosis and actually the ability to get the patient’s story accurately and, you know… So, I probably under diagnosed.” (GPC01)*

In contrast to the health professionals, patients were clear in their belief that CFS/ME should be diagnosed and, at least initially, managed within primary care. When reflecting on the role of their GP, patients wanted to be believed and receive a positive diagnosis. They also wanted their GP to be accessible and actively involved in the longer term management of their condition:

*“Most people who go to the GP can’t understand what’s happening to them. You don’t go to the doctor saying I think I might have ME doctor, can you have a look at me?…You go because there’s something wrong with you… So I think [primary care] is the place where the information should be given out. It should be with your GP, because they’re in a position to explain what might be wrong with you… to offer support” (patient 7)*

Where support was not received, patients reported disengaging from primary care. This highlights the tension between patient’s needs and the barriers to the diagnosis and management of CFS/ME cited by GPs:

*“Why can’t [the GP] say well have you done this, and we need to get you doing this and, I know you’ve had it for a long time but, let’s have a look and see what we can do” (patient 24)*

“*I don’t talk about CFS/ME with the GP… Were she to raise it I would, but I don’t envisage her raising it....I need them to make me feel that they are treating it seriously” (patient 21)*

### Cognitive participation: commitment to the management of CFS/ME in primary care

Only GPs from 10 out of the 21 practices recruited completed the online training, highlighting a low level of commitment to the study. Reasons given for this lack of commitment included the small number of patients with the condition, pressures on time within a consultation to deal with complex problems, and the suggestion that CFS/ME was not a priority as it was not within the Quality and Outcomes Framework (QOF):

*“It’s constantly very busy… there are new QOF things to do, and then also there’s all the changes with commissioning et cetera, we’re to be more involved in that. I think it’s just a workload issue, and unfortunately, there are a lot of clinical areas where many of us are interested, but we just can’t get round to do.” (GPA01)*

*“It’s not top of anyone’s agenda and it’s not realistic to expect it to be… I know my colleagues were disinterested in it because it was not seen… it’s one that you’ll see rarely, therefore, the amount of importance you can give it isn’t that much. It’s not interesting to [GPs] it’s not as important as somebody who’s having a heart attack or trauma or a schizophrenic breakdown or bipolar. So even in the mental health, physical crossover it’s not top of anybody’s agenda” (GPA04)*

These barriers resulted in only 53 % of patients who took part in this study receiving a copy of the information resource. When asked about the mode of delivery of the resource, 47 % of these patients reported that they received the information from their GP within a consultation, 35 % by post, and 18 % from a receptionist or nurse. The packs provided were often incomplete and did not include the information leaflets. GPs stated that this was due to a number of reasons which included misplacing the resource pack, or forgetting what should be provided. This meant that the objective of using the resource pack as a tool to build a positive relationship with the patient within the consultation was not achieved:

*“I wonder did I mail them? I might have done one or two, but I was probably too mean to spend the postage. I might have mailed one or two.” (GPA03)*

*“Initially I thought it was very good but, again, because it’s paperwork and, having to be honest, I’ve no idea where it is and if somebody came in now and asked for it I wouldn’t have it because it’s paperwork. (GPA04)*

*‘I actually forgot there was a DVD… so I didn’t give her the DVD.” (GPA02)*

Patients also recognised a continued lack of commitment to the management of the condition and an inconsistency in knowledge about the study between GPs in each practice:

*‘I was saying to the doctor, I think I might have chronic fatigue and he showed me the pack and said, I’ll get you one of these but he didn’t get me one… he forgot all about it and never got me one… I went back and…about six weeks later I said, did you sort that out? Oh, I’ll get you one, he said, he didn’t get me one… He’s just a bit unorganised, I think he works too many hours (patient 8)*

*I got a letter from the surgery saying, did I want to take part in this study, then, down the line, they don’t seem to know anything about it, which was a bit odd… The GP didn’t have a clue what he was talking about. (patient 11)*

*my pack has nothing in it.... When I went for it… I said I understand you’ve got a pack for me. Couldn’t find them… I had to go back for it. The receptionist went on the hunt for it and found it in one of the offices somewhere and gave me it, but it didn’t have any of this information. (patient 39)*

As a result of not receiving this information, many patients reported disengaging from primary care as this reinforced belief that GPs do not prioritise CFS/ME:

*it was very frustrating to be honest, so I gave up in the end. I was just frustrated really. (patient 11)*

### Collective action: using the resources and working together

When used in the way it was intended, the CFS/ME resource provided the information required for GPs and patients to work in partnership to prioritise symptoms and develop a management plan over a number of consultations. GPs who completed the training said that their knowledge of CFS/ME had improved and they had established a positive relationship with their patients as they felt they now had something to offer:

*“It made me much more confident that there was an appropriate approach… [I have] a more clear idea in my own mind of how to manage it, as opposed to how to treat it, and to be confident that those are sensible things to do, and that they are endorsed. It has made me more willing to use the label, because then I can follow up with saying; and this is what it means, and this is what we’re going to do, and this what’s likely to happen… You have to say, right, I’ll see you again in two weeks’ time… like a lot of things, you spend time to make time; if you invest that time and people get better, then it saves time” (GPA05)*

The video clips on the online training module that showed how a GP can work with a patient within a consultation were particularly valued by a number of GPs:

*I think the online training is good and I think the fact they’d developed it with patients was a good way of doing it. I particularly liked the [video clip] interactions they did about how you review a patient, because I think it just gives you confidence that you’re going through the right motions really” (GPC01)*

Patients with varying severity and time since diagnosis described how the provision of reliable evidence based information meant that their GP was validating their CFS/ME. This enabled them to self-manage their condition:

*I think it’s the thing I keep coming back to, the sort of idea of legitimacy and validation, that there’s a sense of acknowledging the condition which has also forced me to acknowledge it… Yeah, I mean there’s kind of a psychological benefit I think if not a physical benefit… that’s helped me to manage my symptoms” (patient 21)*

However, GPs believed that there were barriers that prevented them from working with their patients. For example, when the training module was completed, GPs reported that they had difficulty remembering the key messages due to limited opportunities to diagnose the condition because it was seen as relatively rare:

*“You see, now I just can’t bring to mind the other diagnostic features that really struck me at the time. I can’t bring them to mind… I was trying to explain to the patient things, and I’m thinking, I’m not sure this is right… (GPA02)*

*“And I know you’ve told me all this [how to use the pack], but I couldn’t remember, that was the thing…A year down the road when you’re diagnosing somebody, I can’t remember, because 47 trillion other people have told us their conditions and how it works for…yeah… maybe a summary sheet would be helpful” (GPC01)*

Moreover, where opportunities arose, GPs and patients reported a lack of time within a ten minute consultation. Patients felt unable to explain the complexity of their condition to their GP. Without the opportunity to relay this information, patients struggled to work with their GP to manage their symptoms:

*I don’t think anybody’s got that time to be able to sit down and spend that amount of time with you. And as well as that you can’t really explain all what we’ve talked about in a ten minute consultation at the doctors. (patient 7)*

*“If we take a primary care approach, we need to have the time… We should be saying, to do good primary care, there need to be enough GPs to have the option to do 15 or 20 minute consultations, or half an hour appointments, when it’s complicated. And it wouldn’t matter what it was for, then; they’d be better at managing diabetes or heart failure, or depression, or anything… If you want people to manage complexity in primary care, you have to give them the time to do it, and then they can do it, actually” (GPA05)*

GPs reported rarely using the information packs with CFS/ME patients who had been diagnosed for some time. Similarly, patients interviewed did not recall receiving the packs:

*When I went in last… [the GP] never mentioned [CFS/ME], no, and I didn’t mention it. I mean, I was asking him other things, and they do only have a certain amount of time, don’t they? (patient 25)*

Furthermore, some GPs said they did not want to discuss CFS/ME with patients who had not mentioned it to them for a number of years as this had the potential to increase their, already stretched workload. This issue was highlighted by the note screening process in one practice, where they excluded patients from the study who had been diagnosed with CFS/ME by another practice as they were not sure if it was an accurate diagnosis. Respondents said that CFS/ME had never been discussed with these patients:

*What I don’t always endorse is people who arrive with a diagnosis that I haven’t made, because I think, I don’t know the basis on which that diagnosis was made… I wouldn’t bring it up” (GPA05)*

Limited referral options were also seen as a barrier to successfully working with patients to manage CFS/ME. Our resources were designed with the aim of increasing the referral of more severe cases of CFS/ME to secondary care services. During the time of our study, a specialist with an interest in CFS/ME retired, and other CFS/ME services were redesigned. GPs therefore remained unsure when they should refer, where to refer, and what the specialist services could offer:

*I guess the one thing that worries me about [managing CFS/ME] in primary care, is being able to have that detailed knowledge about other resources and where to refer… And it’s a bit like dementia, it just changes all the time, it’s actually very hard to keep tabs on that.” (GPA02)*

*“I’d like to know the specific details, in terms of, the waiting time for an assessment, and the referral criteria. Because, although we’ve been told it’s there, I don’t recall getting the detail where it can make a difference in terms of informing a patient, and what you do with a patient in the meantime.” (GPA01)*

### Reflexive monitoring: evaluating the impact of the training and resources

The study allowed GPs and patients to reflect on the management of CFS/ME in primary care. Patients who had not received the patient resource from their GP were sent the pack by the research team before interview. Some patients reported a noticeable improvement in their GP’s knowledge of CFS/ME following the training:

*I think [the GP] has been helping me a lot more in the past 6 months. She’s got better at helping me when I go in there… she’s referred me to the pain clinic.... And when I refer to things instantly there’s no vacant look on her face. Like if I’d have said to her before I lose the thread of what I’m saying in a sentence, she’d say what’s that got to do with anything? Now she immediately knows that that’s one of the things that’s part of the condition. (patient 7)*

The resources had a positive impact on the patient’s understanding of CFS/ME. The DVD case studies were seen as particularly important in helping patients and carers to understand that others shared their experiences, and the format allowed those who found it difficult to read to access the information. As a result of this information some patients felt that they needed to visit their practice less frequently:

*[I visit] less frequently because I feel that…because I was so desperate because I had all these symptoms and I’m going back all the time with different symptoms… But now it’s just easier to accept that I might have all these symptoms because I’ve got [CFS/ME] instead of trying to look for something in each symptom. (patient 8)*

*[The DVD helped] me see other people going through exactly the same thing… Looking the same and feeling the same … So you know it’s not just me because of being lazy, not pushing myself enough, yeah… (patient 8)*

The resources were also reported to have had an impact on the friends, family and colleagues of the patients interviewed. In some cases, the provision of evidence based information improved relationships and strengthened support networks:

*I also watched the bits [of the DVD] again when my friend was watching it, yeah… he said it was really interesting and it’s really helped him understand… My daughter is also much kinder to me since she’s got the information… she needed to see it from an outsider’s point of view and so it has helped, yeah (patient 8)*

*There was one particular set of experiences [on the DVD] from a carer that I think would be very helpful for other people in a kind of care role (patient 21)*

*one woman who went back to work this summer found them very helpful; and I think she found it helpful because it made her condition comprehensible. (GPA05)*

Patients and GPs stated that the resource pack would be of greatest benefit to newly diagnosed patients. However, a number of patients who had the condition for a number of years reported that a comprehensive pack of information allowed them to consolidate their knowledge and sometimes learn something new:

*I think that’s okay when you’ve been diagnosed, but for somebody like me that’s had this problem for [a long time] I don’t get anything from that… but it’s good to keep referring to this every now and again. Had I had that booklet right at the beginning I’d have thought God, I’ve just won the lottery. There’s a lot of information in there that’s very helpful. (patient 7)*

*there were certainly some of the different therapies for things that I hadn’t heard of or had forgotten had I heard of them. (patient 21)*

Although GPs and patients reported that the training and resources could have a positive impact on the diagnosis and management of CFS/ME in primary care, it is important to consider the limiting effects of the barriers to implementation described during the interviews. In order to overcome these barriers, GPs and patients suggested that the resources should be made available online and therefore accessible to all. An evidence based source of information was welcomed as there are currently issues with identifying reliable information on the internet:

*Information online would be useful… Well you’d be able to get more information quicker and it’d be proper reliable information rather than just people’s stories. (patient 18)*

*“what I’d like is that you had all that information on the website and I can give people the address… I can say to patients, look, this is…I know this is a good website and the information, I believe is up to date, you know, I’m totally with you on the condition. Obviously, if they don’t have internet access, I can print them off the booklet, but printing things off, bit by bit, off one PDF is just not a goer really, as far as I’m concerned.” (GPC01)*

*“I think our new computer system probably would allow for some kind of prompt… so I think that is possibly the way to go, is to say, oh, you’ve typed in CFS; here is a link to some resources on a website that might be useful.” (GPA05)*

Some GPs and patients suggested websites that they believed would be a useful for the resources to be linked to in order to be easily accessible. These included NHS Choices website (patient 18), YouTube or HealthTalk online (GPA05) and RCGP (GPA04). However, some patients were concerned that by placing the resources online, GPs would be ‘let off’ managing the condition in primary care and skeptical attitudes would continue:

*“the problem with putting resources on the internet is then that you’re cutting out the GP and then the GP is again kind of getting away with not having any involvement. And they should be made to take it seriously do you know what I mean? You’re letting them get away with it scot free then. That’s just saying well, brush it under the carpet like it’s always been.” (patient 24)*

Patients therefore reported that they wished to bring information from the internet to the consultation in order to gain a diagnosis from a health professional. Patients had done this in the past, and GPs welcomed this where information was from a reliable source:

*“[In the past] when I printed the information off from the internet I wasn’t sure that that’s what I had and I was just clutching at anything to find out what was wrong with me. But if a GP [confirms it] and says, yeah, this is wrong with you, you can relax and learn to accept it and cope with it.” (patient 8)*

*“Oh, I’m all for patients being able to access reliable information. I mean, we’ve quite a few that come in and tell us already, I've read this, I’ve read that, et cetera… If patients come in and they’ve looked…I mean, that makes my life easier, I’d much rather people went on the internet, but, obviously people don’t know how to quality assure what they’re seeing, so I think there needs to be a way of badging it…” (GPC01)*

A second recommendation to increase the impact of these resources was to call for greater investment in secondary care services. For example, most GPs interviewed in this study reported that training highlighted the complexity of the condition. They therefore believed that it would be more appropriate for CFS/ME to be managed by a specialist service:

*“I think you need more backup… you say; oh, you need graded exercise, you need a bit of physio, and you think; well, where the bloody hell are you going to get it? Sort of an ME specialist nurse or an ME specialist…even one for the whole of Manchester or the North West wouldn’t be a big deal because they could be accessed by phone. So, yes… if I had a specialist nurse, like I’ve got an MS specialist nurse, if I had an ME specialist nurse and they could cover hundreds of practices because there’s not a big uptake, that you could tap into their expertise and say; look, is there a day centre where they tend to get together or could they go to the…what’s that course? Could they go to a back to work course, at the moment they’re just feeling much better and when is an appropriate point for them to go to the low activity back to work?” (GPA04)*

Patients also wanted more access to specialist services, with some recognising that GPs didn’t have the time to manage their condition:

*“I think the GPs are too busy and there’s too much going on for them to build up that wealth of knowledge… I’m quite happy to have a diagnosis from a specialist service, but what I would like to see is that the GP will refer you.” (patient 39)*

## Discussion

This study provides further insight into the reasons why many GPs do not engage with the diagnosis and management of CFS/ME in primary care. Normalization Process Theory (NPT) was employed to examine the extent to which newly developed CFS/ME training and resources can be implemented and ‘normalized’ under routine conditions. Although GPs and patients reflected positively on the potential or experienced benefits of the CFS/ME training and resources (reflexive monitoring), they recognized that there are currently too many barriers to commit to managing a complex condition such as CFS/ME in primary care (coherence). For example, time pressures and competing priorities meant that some GPs failed to engage with the training module (cognitive participation). When the module was completed, many GPs stated that it was not feasible to retain even the key messages as they saw so few patients with the condition. Furthermore, half of the patients recruited to the study did not receive the information pack from their GP. When they did it was often incomplete, sent in the post, and GPs did not work with patients to discuss the materials (collective action). The resource was therefore not implemented in the way that was preferred by patients nor as had been intended when it was developed by the research team.

### Comparisons with existing literature

Previous literature has highlighted a lack of GP confidence in the diagnosis and management of CFS/ME [7, 15] and a need for training [3, 16]. However, despite access to a CFS/ME training module, the majority of GPs in this study were still unable to successfully implement CFS/ME resources in their surgery. Our findings are therefore consistent with previous studies of innovative models for long term condition management in primary care which have shown the difficulties that exist when trying to modify established roles in order to improve care [17]. Integrating new training and resources for a complex, low prevalence, medically unexplained condition into routine practice can clash with existing norms and beliefs, including scepticism around CFS/ME and the belief that this condition should be managed in secondary care [3, 18]. In order to tackle the beliefs that act as barriers to the implementation of the CFS/ME resources, Bayliss et al. [2] suggest a need for the inclusion of this condition on the medical school curriculum. The current study also exposes the tensions that are inherent in research which ‘denormalizes,’ existing ways of working in order to introduce new ones [18]. This is especially the case when GP surgeries were already experiencing significant change at the time of the study, including the introduction of Clinical Commissioning Groups (CCGs) [19].

### Limitations

The study included interviews with a relatively small number of patients and GPs. This was because it was difficult to engage practices and GPs in research on CFS/ME. Although practices were offered a fee to take part in the study and an additional payment for each GP to complete the online training module (which could also be counted as CPD points) only 21 practices across North West of England engaged in the study. However, it is important to note that the launch of the study coincided with the change over from Primary Care Trusts to CCGs. As a result many practices were changing their IT systems and may not have had the time to engage with this study.

In the practices that did participate, most GPs failed to implement the resources provided. GPs did not see CFS/ME as a major part of their workload and also experienced pressure to focus on conditions incentivised as part of the Quality and Outcomes Framework (QOF), a pay-for-performance scheme that financially rewards GP practices for achieving a number of clinical and organisational indicators [6, 20].

### Recommendations for practice

Barriers to the implementation of CFS/ME resources in primary care means that many patients will be left without the support they need to manage their symptoms and work towards recovery. The training in this study highlighted the complexity of the condition which led GPs to believe that it would be more appropriate for CFS/ME to be managed by secondary care. Patients similarly wanted more access to specialists. However, NICE guidelines emphasize the role of primary care in the management of CFS/ME and specialist services may not be commissioned by all CCGs. It is hence evident that a disconnect exists between recommendations and practice and it is not clear where the responsibility for the management of CFS/ME lies.

Patients living with medically unexplained conditions are increasingly turning to the internet to find information on their condition [21]. However, although the internet can be enlightening, it can also misinform patients about their chronic condition [21]. Our findings highlight the need for a reliable, easily accessible online evidence based resource on CFS/ME. This information could be shared with family, friends and colleagues in order to reduce the stigma that can surround the condition, and build support. [3] It could also be used to inform, and prepare for, doctor-patient interactions [3, 21]. The GPs in this study supported the idea that patients could bring information to the consultation, as long as it was from a reliable source. GPs also suggested that a link to the training module should be provided online for those with an interest in CFS/ME. The online training was made available on the RCGP website to all GPs and between May 2012 and February 2014, 1065 unique users accessed the course, suggesting that it may be more effective for resources to be directly accessible to patients and health professionals on the internet rather than using the GP as a conduit as we did in the current study.

## Conclusion

Our analysis demonstrated that when used correctly the information included in the resource for the diagnosis and management of CFS/ME was beneficial to both patients and GPs in the diagnosis and management of CFS/ME. However, this feasibility study suggests that it is not possible to for GPs to implement this resource into UK primary care due to limits to GP time, and the low priority placed on low prevalence, contentious, hard to manage conditions.

While barriers to good quality care remain, there is a need to support CFS/ME patients to access evidence based information outside primary care. Both GPs and patients in this study suggest that the development of an online resource that provides instant access to advice from CFS/ME specialists will help to reduce the emotional and physical burden currently experienced by CFS/ME patients who are either left with no support or on long waiting lists to access secondary care services. By presenting information on CFS/ME in the public realm, patients will be empowered to overcome the barriers that currently stand in their way to gain a diagnosis and manage their symptoms.

### Availability of data and materials

Please note that all the data supporting our findings is contained within the manuscript.

## Additional files

Additional file 1: METRIC topic guide patient evaluation. (DOC 61 kb) Additional file 2: METRIC topic guide practitioner evaluation. (DOC 71 kb)
