# Supplementary material for: Implementing resources to support the diagnosis and management of Chronic Fatigue Syndrome/Myalgic Encephalomyelitis (CFS/ME) in primary care: A qualitative study
Source: BMC Fam Pract. 2016 Jun 4;17:66. doi: 10.1186/s12875-016-0453-8 (PMC4893302; doi:10.1186/s12875-016-0453-8)
Supplement: Additional file 1: — METRIC topic guide patient evaluation. (DOC 61 kb) [file 12875_2016_453_MOESM1_ESM.doc]

#
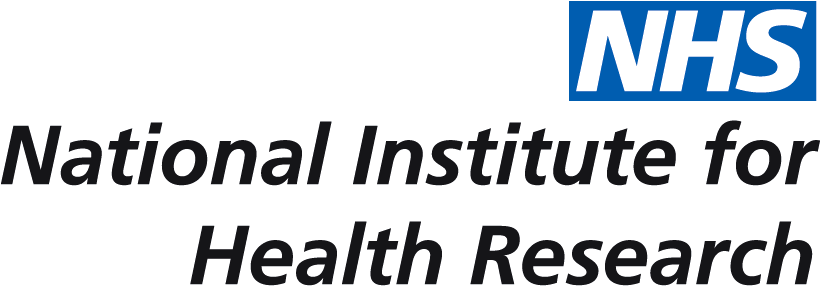
METRI˚C

**ME Education Training & Resources in Primary Care**

TOPIC GUIDE – Patients

The aim of this interview is to examine your experiences of seeking help for problems to do with your CFS/ME from your GP and/or practice nurse.

Could you please tell me about your background (age, ethnicity, employment status, duration of illness etc).

1. Could you start by telling me about your experience of seeking help from your GP/PN for your CFS/ME?
   1. Had you received any advice/information in the past on the management of CFS/ME from your GP or Practice Nurse?
   2. Have things changed recently?
2. Can you tell me about your current experience of seeking help for CFS/ME and with any other problems (in the past 6 months)?

- How often do you consult your GP and/or Practice Nurse? Has this changed recently?
- Do you talk about your CFS/ME?
- Do you tend to see one GP preferentially? Why? What makes you choose one GP over another?
- What do you think your GP/PN understands by CFS/ME and how it affects you? Has this changed recently? If so, in what way?
- What sort of advice have you been given on how to manage your condition?
- Have you been given any information^*^ from your GP or PN about CFS/ME? What sort of information?
- Have you been referred to any other services? Which? Can you tell me about the advice and help you received from that service?
- What else do you think your GP or PN should do to help you manage your condition?

1. You mentioned that your GP/PN gave you some information^*^ about CFS/ME.

- How was this information given to you?
- Did you get the whole pack at once or in stages?
- How did you find the leaflets?
  - CONTENT and PRESENTATION
  - USEFUL and RELEVANT
- Did you show the leaflets to anyone else? Did you partner/family/carer find them useful?
- Did you discuss any of the leaflets with the GP?
- Did you use the patient activity diary?
- How did you find the DVD?
  - CONTENT and PRESENTATION
  - USEFUL and RELEVANT
- Did you show the DVD to anyone else? What did they think of it?
- Was there anything in any of the resources you didn’t like/didn’t find useful/not relevant? Why?
- Is there anything about the information you received that you would like to change?
  - CONTENT and PRESENTATION
  - RELEVANCE
- Is there anything about the way the resources were given to you that you didn’t like? How could this have been improved?
- If BME patient: Did you find the resources were relevant to your specific needs? How could they have been improved?
- Do you think the GP is the best person to give you this information? How would you feel if this information had been available on the internet? Would you have accessed this?

1. Finally, what do you think have been the main benefits of your participation in this study? Have there been any disadvantages?

* show them patient resource pack at question 3.
