# Supplementary material for: Implementing resources to support the diagnosis and management of Chronic Fatigue Syndrome/Myalgic Encephalomyelitis (CFS/ME) in primary care: A qualitative study
Source: BMC Fam Pract. 2016 Jun 4;17:66. doi: 10.1186/s12875-016-0453-8 (PMC4893302; doi:10.1186/s12875-016-0453-8)
Supplement: Additional file 2: — METRIC topic guide practitioner evaluation. (DOC 71 kb) [file 12875_2016_453_MOESM2_ESM.doc]

#
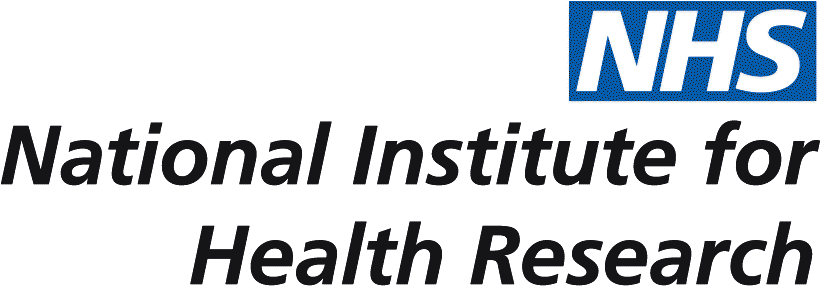
METRI˚C

**ME Education Training & Resources in Primary Care**

TOPIC GUIDE – GP interview

The aim of this interview is to examine your experiences of the training you received about CFS / ME as part of the METRIC study, and how you have found working with your patients with CFS/ME.

Could you start by telling me a bit about your practice (e.g. demographics, list size, practice team, etc)?

1. Could you tell me why you agreed to participate in the METRIC study?
2. Could describe your experience of CFS/ME before you received the training? It would be helpful to tell me about any patients you previously managed with the condition (also explore any personal experience of CFS/ME – self, family).
3. Could you tell me if your view of CFS/ME has changed? And if so, in what way? Do other GPs in your practice hold the same views?
4. Can you tell me about your experience of the training itself?

- Did you participate in the face-to-face training or use the on-line module?
- If face-to-face - Who participated in the training? How was this for the practice?
- If on-line – why? Did all GPs in your practice complete this? If not, why?
- What was particularly useful and why?
- Was there any part of the training that you didn’t find useful? Why? (present a list of the different sections to see which they found most/least helpful)
- Were the video clips useful? Did you watch all of these? What did you like/dislike about these?
- Did you learn anything new? (ask them to describe anything they were not aware of before the training).
- How confident do you now feel about making the diagnosis of CFS/ME? Can you describe how you would make a diagnosis?
- Do you think you have diagnosed anybody with CFS/ME since you did the training? Did you use the resources to aid this diagnosis? How?
- Are you more or less likely to make the diagnosis now you have completed the training?
- How do you feel about managing patients with CFS/ME? Can you tell me how you would manage patients?
- Will you do anything differently as a result of participating in the training?
- Has the training helped you in other areas of your work?
- Did you use the computer templates? Were these helpful?

1. What are your thoughts about the patient resources we have developed?

- Did you look through them?
- Have you used the resources with CFS/ME patients? How?
- Do you have the information sheets saved on your desktop? Are they easy to access/print off?
- How useful do you think the information sheets are? Did you find any particularly helpful?
- Did you look at the DVD? What do you think of this? Was it useful?
- Are there any negative effects of these resources?
- Is there anything about the resources that you would like to change?
- How did you use the resources? Did you give out complete packs or encourage patients to come back over a number of consultations to go through different info sheets/monitor progress. Did these patients require extended consultations?
- Do you think that primary care is the best place for these resources to be used?

1. Did you use the activity diary? How? Did it aid diagnosis? If not, why did you not use this? Do you think it could be helpful?
2. Have you referred more patients to secondary care as a result of this training?
3. How do you feel about diagnosing and managing CFS/ME in black and minority ethnic groups?

- Do you think the resources are useful for CFS/ME patients from BME communities? Or do they need modification?
- Has the training helped you in the diagnosis and/or the management of CFS/ME in BME patients?
- Has your approach to patients from BME communities changed as a result of the training / resources?

1. What do you think have been the main benefits of your participation in METRIC? Have there been any disadvantages?
2. Finally, it has been difficult to engage practices in this research. Why do you think some GPs may not want to take part? How do you think we can encourage GPs to engage with the diagnosis and management of CFS/ME?
3. What do you think the idea of adapting this patient information for an online resource, for patients to access outside primary care?
4. Any other comments? Thank you.
